# Laparoscopic fluorescence image-guided photothermal therapy enhances cancer diagnosis and treatment

**DOI:** 10.7150/ntno.28585

**Published:** 2019-01-29

**Authors:** Mohan Singh, Elham Nabavi, Yu Zhou, Maria Elena Gallina, Hailin Zhao, Pakatip Ruenraroengsak, Alexandra E. Porter, Daqing Ma, Anthony E. G. Cass, George B. Hanna, Daniel S. Elson

**Affiliations:** 1 Hamlyn Centre for Robotic Surgery, Imperial College London, London, UK SW7 2AZ.; 2 Department of Surgery and Cancer, Imperial College London, London, UK SW7 2AZ.; 3 Department of Chemistry, Imperial College London, London, UK SW7 2AZ.; 4 Department of Materials, Imperial College London, London, UK SW7 2AZ.

**Keywords:** Photothermal therapy, image-guided therapy, fluorescence endoscopy, near infrared fluorescence, theranostics, surgical technology, gold nanoparticles

## Abstract

Endoscopy is the gold standard investigation in the diagnosis of gastrointestinal cancers and the management of early and pre-malignant lesions either by resection or ablation. Recently gold nanoparticles have shown promise in cancer diagnosis and therapeutics (theranostics). The combination of multifunctional gold nanoparticles with near infrared fluorescence endoscopy for accurate mapping of early or pre-malignant lesions can potentially enhance diagnostic efficiency while precisely directing endoscopic near infrared photothermal therapy for established cancers. The integration of endoscopy with near infrared fluorescence imaging and photothermal therapy was aided by the accumulation of our multifunctionalized PEG-GNR-Cy5.5-anti-EGFR-antibody gold nanorods within gastrointestinal tumor xenografts in BALB/c mice. Control mice (with tumors) received either gold nanorods or photothermal therapy, while study mice received both treatment modalities. Local (tumor-centric) and systemic effects were examined for 30 days. Clear endoscopic near infrared fluorescence signals were observed emanating specifically from tumor sites and these corresponded precisely to the tumor margins. Endoscopic fluorescence-guided near infrared photothermal therapy successfully induced tumor ablations in all 20 mice studied, with complete histological clearance and minimal collateral damage. Multi-source analysis from histology, electron microscopy, mass spectrometry, blood, clinical evaluation, psychosocial and weight monitoring demonstrated the inherent safety of this technology. The combination of this innovative nanotechnology with gold standard clinical practice will be of value in enhancing the early optical detection of gastrointestinal cancers and a useful adjunct for its therapy.

## Introduction

Esophago-gastric cancers often grow indolently and are diagnosed late, resulting in only a third of patients being suitable for definitive treatment and with less than 20% being alive at 5 years. Endoscopy is universally accepted as the gold standard investigation for the diagnosis of esophago-gastric cancers. However, esophago-gastric cancers may be missed at endoscopy in 8% of patients who are subsequently diagnosed with cancer^1^, ^2^. Accurate mapping of early cancer and pre-malignant lesions directs the precision of endoscopic therapy either by resection or ablation^3^. The adequacy of endoscopic therapy involves a balance between removing the entire lesion for cancer clearance but without compromising much of the healthy surrounding tissues to avoid strictures and other complications. Clear margins are essential for a curative approach. Early detection involves detection of gastrointestinal (GI) tumors before they breach the muscle layer (muscularis propria). As some tumors spread along a submucosal plane, this may preclude any apparent mucosal abnormalities, and thus can be missed by standard white light endoscopy.

Gold nanoparticles (GNPs) have attracted much interest in biomedical applications due to their ability to be functionalized with fluorophores, peptides and receptors for tumor targeting and surgical imaging, whilst their high optical absorption of near infrared (NIR) light which can induce tumor hyperthermia and thus effect photothermal therapy. Published studies on the application of GNPs as enzyme-activated contrast probes in the detection of gastrointestinal (GI) tumors have been fraught by barriers such as the targeting of cancer moieties which also co-exist within normal physiological tissues and a reliance on the pH of the tumor microenvironment^4^, which is complex, variable and unpredictable. As such these nanoprobes become less reliable due to non-specific targeting and tumor heterogeneity.

Other forms of molecular nanoprobes for tumor localization have been utilized as alluded to in recent systematic reviews of GNPs for cancer theranostics^5^-^7^ including single-photon emission computed tomography-computed tomography SPECT-CT^8^, computed tomography (CT)^9^, ^10^ and positron emission tomography (PET)^11^-^13^. However their application in cancer imaging in humans is limited as they lack sensitivity, involve nephrotoxic contrast and high radiation doses. MRI^14^, optical coherence tomography (OCT) 15 and magnetic iron oxide based nanoprobes^14^ are unsuitable as these modalities are incongruous with well-established clinical investigations for suspected GI cancer and thus would fall short of gaining widespread acceptance over conventional endoscopy.

The integration of a nanoprobe with fluorescence endoscopy facilitates the introduction of effective minimally invasive theranostics into clinical practice by utilizing a conventional clinical device. This approach will mitigate barriers to clinical adoption. Endoscopy is utilized in diagnosis of GI cancer worldwide because of its high sensitivity and specificity^16^, ^17^. By delivering GNPs in parallel with fluorescence endoscopy, even subtle GI lesions would be rendered optically distinct, thus facilitating image-guided biopsies and formulating a clear target for the synchronous photothermal therapy of these lesions. This is because fluorescence endoscopy would be able to detect the emission of optical feedback emanating from the accumulation of GNRs even from subtle submucosal or early cancers.

This study utilizes the multifunctional properties of gold nanorods (GNRs) in an endeavor to establish a novel endoscopic approach for *in vivo* upper GI cancer theranostics. It harnesses both active and passive tumor targeting of *in vivo* esophageal adenocarcinoma tumors in BALB/c nu/nu immunodeficient mice receiving GNRs functionalized with an optical fluorophore (Cy5.5) modified with anti-EGFR antibody together with image-guided NIR irradiation to enhance live cancer site-specific fluorescence and hyperthermia. The methods incorporate the use of a simple, low cost and reproducible design which can complement endoscopy by enhancing real-time cancer diagnosis with fluorescence imaging and further develops simultaneous, rapid and highly effective tumor photothermal therapy. This work additionally evaluates the safety of multifunctional GNRs as a treatment modality *in vivo*, as a validation of its feasibility for clinical application.

As the ideal route of GNR delivery for optimal photothermal tumor ablation is still undetermined^18^-^20^, both intravenous (IV) and intratumoral (IT) GNR administration routes were assessed to attempt to achieve complete clinical remission of tumors by the end of the study period, as examined by conventional histopathology. IT administration of GNRs is a feasible solution to overcome the molecular heterogeneity within tumors and is thus clinically relevant. Endoscopic IT injection of GNRs followed by photothermal therapy (PTT) could also induce tumor debulking, being thus applicable in the palliation of symptoms arising from large or inoperable GI and hepatic tumors. This amalgamation of innovative nanotechnology with gold standard clinical practice aims to be valuable in the optical detection of early GI cancer whilst efficiently augmenting the surgical armamentarium for treatment.

## Materials and Methods

The structure of reporting the *in vivo* methodology and results here is in line with the ARRIVE (Animal Research: Reporting of *In Vivo* Experiments) guidelines which are currently endorsed by scientific journals, major funding bodies and learned societies^21^.

### Ethics

Ethical approval was sought under the Animals (Scientific Procedures) Act 1986 and was granted by The United Kingdom's Secretary of State under a small animal project license (PPL number 70/7996).

### Synthesis and functionalization of GNRs

GNRs were fabricated using the seed-mediated method described by Murphy *et al.*^22^ (see Supplementary section). CTAB molecules on the surface of GNRs were replaced by carboxyl PEG. Subsequent EDC/NHS chemistry was used to functionalize GNRs with a fluorophore (Cy5.5) modified with an anti-EGFR antibody. Functionalized GNRs were used in order to actively enhance specific tumor targeting efficiency rather than relying exclusively on the preferential passive accumulation of nanoparticles in tumors as a result of EPR. Further details are provided in the supplementary methods section. Functionalized GNRs were imaged using TEM (TEM JEOL JEM-2000FX, Tokyo Japan) and the average length was 43.7 ± 6.3 nm with an average width of 11.6 ± 1.2 nm, yielding an aspect ratio of 3.9 ± 0.6 nm. Epidermal growth factor receptor (EGFR), found on the surface of cells, is commonly upregulated in a variety of epithelial malignancies, including esophageal, gastric and colon adenocarcinomas. EGFR inhibitors such as Cetuximab are indicated in the treatment of metastatic colorectal cancer, metastatic non-small cell lung cancer and head and neck cancer. They have been explored in patients with upper GI cancers, and it is apparent that distal esophageal and esophago-gastric junctional (EGJ) tumors may be more sensitive to EGFR blockade than distal gastric adenocarcinomas^23^. In esophageal cancer, immunohistochemistry (IHC) proven EGFR overexpression is very common, occurring in approximately 80% of patients with adenocarcinoma and squamous cell carcinoma^24^.

### UV-VIS Spectrophotometry

**Figure 1:**
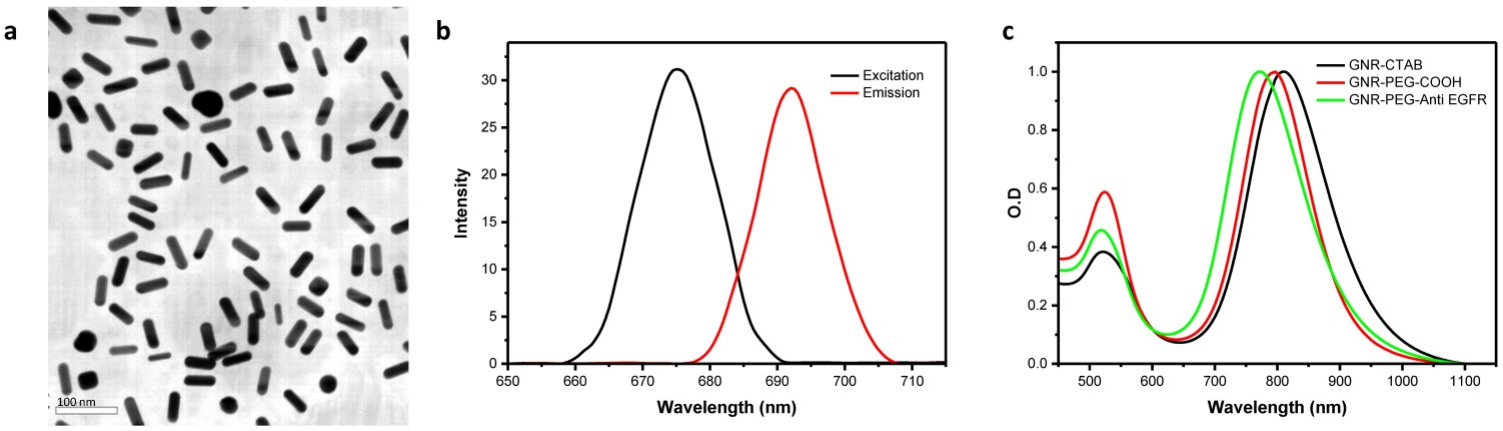
** (a)** TEM image of mutlifunctionalised PEG-GNR-Cy5.5-Anti-EGFR-antibody. **(b)** Optical absorption and emission. **(c)** Fluorescence spectra.

The optical density and light absorption profile of GNRs was measured using a Lambda 25 UV-Vis spectrophotometer (Perkin Elmer, USA), while fluorescence spectra were measured using a Fluorolog-3 478 fluorometer (HORIBA, Jobin Yvon, USA). Pre- and post-functionalization spectra were measured. ζ-potential was calculated using a Zetasizer Nano ZS90 DLS System (Malvern Instruments Ltd., Worcestershire, UK). The results are shown in Figure 1 and Supplementary Tab. S4.

### Characteristics of the functionalized GNRs used in *in vitro* and* in vivo* studies

Multifunctional PEG-GNR-Cy5.5-Anti-EGFR-antibody GNRs were fabricated which by design had an excitation peak at 675 nm and a corresponding emission peak at 692 nm (Fig. 1). The SPR of final solution of PEG-GNR-Cy5.5-anti-EGFR-antibody was measured to be 808 nm, which corresponded to an OD_λ = 808_ of 26.4 (of the undiluted sample) and was not affected by functionalization (Fig. 1). The concentration of this solution was 5.50 nmols/l, or 5.50 nM. The change from a strongly cationic to slightly anionic charge following functionalization (zeta potentials recorded in Supplementary Tab. S4) meant there was good replacement of CTAB ligands on GNRs. We observed good stability and dispersability of the PEG-GNR-Cy5.5-anti-EGFR-antibody in both water and organic solutions.

### Human esophageal adenocarcinoma cell line and culture

FLO-1 human esophageal adenocarcinoma cells were used both for immunohistochemistry and establishing a tumor xenograft in mice. FLO-1 cells have been verified as a true human esophageal adenocarcinoma cell line and are recommended for research on esophageal adenocarcinoma^25^. FLO-1 cells were established from a primary distal esophageal adenocarcinoma in a 68-year-old Caucasian male in 1991. They are of epithelial origin and have an adherent growth pattern^26^. FLO-1 cells were passaged and incubated at 37°C in humidified air with 5% CO_2_ and maintained in a state of logarithmic growth. The culture medium was Dulbecco's Modified Eagle's Medium (DMEM) - 4500 mg glucose/ml with the addition of 10% Fetal Bovine Serum, 2 nM L-Glutamine Solution Bioxtra 200 mm and 100 U/ml Penicillin + 100 mg/ml Streptomycin. Het-1A cells (a healthy, non-tumorigenic human squamous esophageal cell line) were also used for *in vitro* immunohistochemistry comparison of functionalized GNR binding. The HET-1A cell line was obtained from ATCC and was originally derived in 1986 from a 25-year-old black male from autopsy tissue from an area of normal esophageal epithelium by transfection with plasmid pRSV-T consisting of the RSV-LTR promoter and the sequence encoding the simian virus 40 large T-antigen. The HET-1A cell line was cultured in BEGM (bronchial epithelial growth medium; Lonza) at 37°C and 5% CO_2_.

### Immunohistochemistry of cells incubated with GNRs

For *in vitro* fluorescence microscopy, we stained FLO-1 cell nuclei with DAPI and purchased fluorescent anti-EGFR antibody which was added to the cells in culture medium. FLO-1 cells were fixed in paraformaldehyde in 0.1 mol/l PBS solution. Cells were then incubated in 10% normal donkey serum in 0.1 mol/l PBS-Tween 20 and then incubated overnight with rabbit anti-EGFR (1:200, Abcam) followed by incubation with donkey anti-rabbit secondary antibody for 1 hour. Cells were also incubated overnight with our functionalized GNRs. The slides were counterstained with nuclear dye DAPI and mounted with VECTASHIELD Mounting Medium (Vector Laboratories, Burlingame, CA). Cells were examined using an Olympus (Watford, UK) BX4 microscope using a 20x objective.

**Figure 2:**
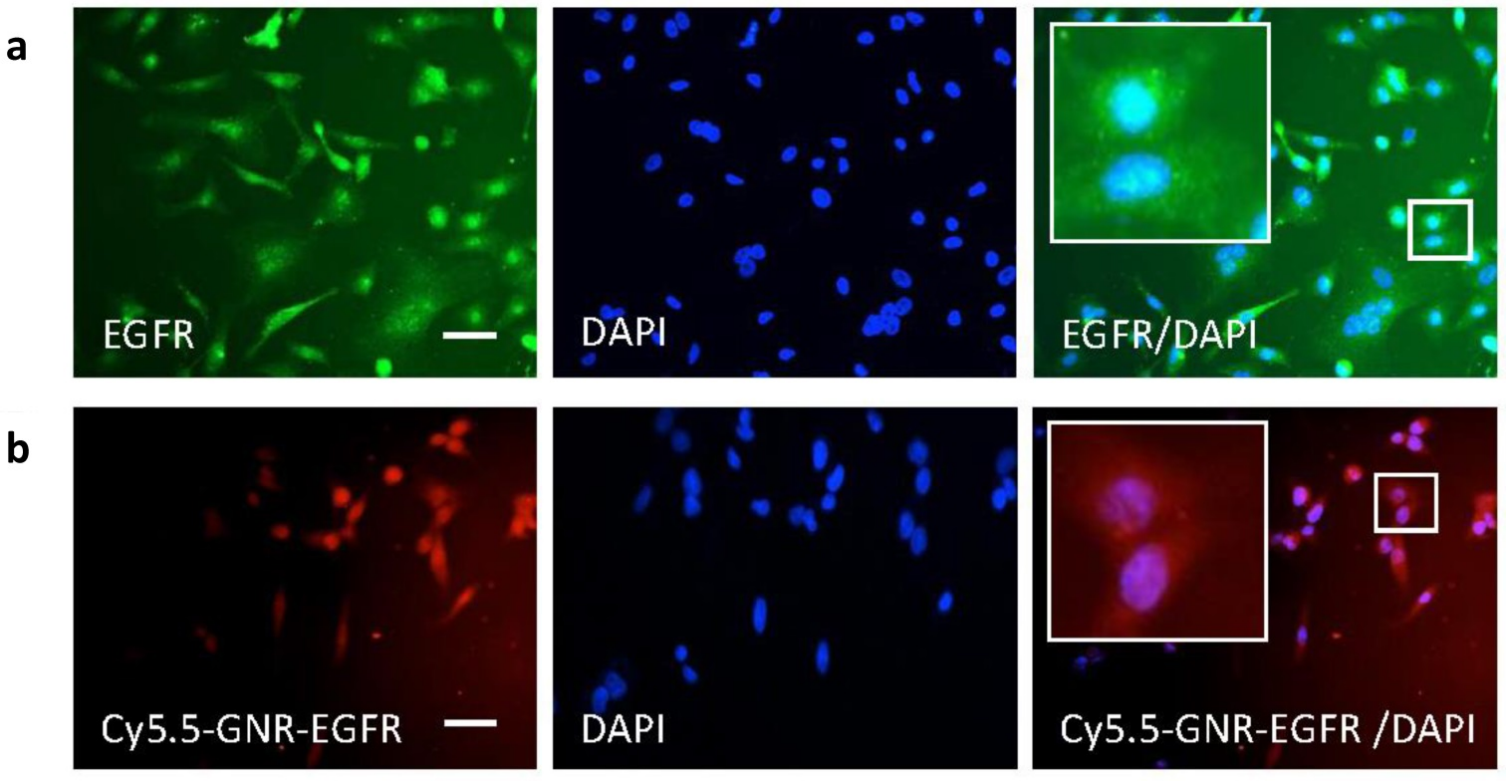
FLO-1 cell immunohistochemistry cell imaging results. **(a)** Blue nuclear DAPI staining and green anti-EGFR antibody fluorescence of FLO-1 cells. The merged EGFR/DAPI image demonstrates binding of anti-EGFR antibody around the nuclei. **(b)** FLO-1 nuclei with DAPI and fluorescence from the Cy5.5-GNR-anti-EGFR-antibody showing in red. Merged Cy5.5-GNR-EGFR/DAPI fluorescence microscopy images demonstrating the binding of FLO-1 cells to antibody- and fluorophore-conjugated GNRs. Scale bar = 50 µm.

Results from immunohistochemistry experiments shown in Fig. 2 illustrated that FLO-1 esophageal cancer cells (with blue stained nuclei) overexpressed the EGFR receptor on their cell surface as they took up free anti-EGFR antibody (in green). These cancer cells were thus targeted for *in vivo* experiments by subsequently conjugating PEGylated GNRs with anti-EGFR-antibody (along with the optical fluorophore Cy5.5), and the results are shown in Fig. 2. This demonstrated that Cy5.5-PEG-GNR-EGFR was internalized by FLO-1 esophageal adenocarcinoma cells (red fluorescence) and was perinuclear, with no fluorescence signals coming from isolated, untargeted or free-floating fluorophores. This binding was not observed with Het-1A cells (a healthy, non-tumorigenic, squamous esophageal cell line^2^. This is likely to be because healthy human esophageal cells like Het-1A show negligible EGFR expression as shown both *in vitro^3^* and using Western Blot techniques.^4^ The FLO-1 human esophageal adenocarcinoma cell line was shown in our immunofluorescence studies and also in Hartmans et al.^5^ to demonstrate an over-expression of EGFR, which naturally became the source of targeting esophageal tumors by conjugating anti-EGFR-antibodies onto our GNRs. Western Blot and flow cytometry techniques to confirm the level of expression level of EGFR would be warranted in future studies to verify results obtained through our immunofluorescence studies.

### *In vivo* validation of fluorescence imaging and photothermal therapy

*In vitro* and *in vivo* pilot studies were conducted to determine the propensity for tumor growth, the optimal GNR dose, laser fluence and duration of irradiation. Further details of the *in vitro* work and diagnostic tests is found in the supplementary methods and results.

### Experimental animals

8-9 weeks old male immunodeficient BALB/c nu/nu mice were obtained from Charles River UK Ltd. (Kent, England). They underwent an acclimatization period after arrival. Each animal was housed individually in a cage (polycarbonate, open on the top and covered with steel wire lid, 29.0 x 11.0 x 11.0 cm deep) and fed with conventional chow [RM1 (P), Special Diets Services, England] and sterile water *ad libitum* while being maintained under controlled conventional environmental conditions at 20 ± 2ºC ambient temperature and 54 ± 2 % humidity, with 12/12 hour light/dark cycles. Food, sawdust and nesting material were autoclaved.

The inoculation area was cleaned and sterilized with ethanol and/or iodine solutions. A pipette was used to mix the cells with their medium and a 26-gauge needle was used to draw the cells into a 0.5 ml syringe. Under manual restraint, 10^6^ - 10^7^ FLO-1 cells in 100 µL culture medium were injected subcutaneously (s/c) at one site, usually into either flanks (or the back) of the mice. The animals were suitable for either treatment or control once their tumors reached a minimum size of 5 mm in any axis. Tumors were measured regularly with digital calipers (Electronic Vernier Caliper, Sealey Group, Bury St. Edmunds, Suffolk, UK) by two doctors who both agreed on the measurements. Tumors were measured in two axes (using calipers in three directions; maximum length, width and depth). All animals were culled humanely if/when the tumors exceeded 12 mm in diameter in two axes. Confirmation that the tumors were appropriate was obtained through punch biopsies and histological evaluation. This was consistent with active and well-established adenocarcinoma of an alimentary tract origin.

### Sample Size

A pilot study was initially conducted with 15 mixed SCID and BALB/c mice, with a further 30 BALB/c mice used in the experimental and control arms. Of these 30, three mice with tumors were used for complementary studies and seven others were used in control studies. After completing the pilot, control and complementary studies, 20 of these 30 mice were randomized within the main study assessing tumor regression after PTT to receive either IT or IV GNRs, thus there were ten mice receiving each method of GNR administration. Control mice (with tumors) received either IV GNRs, IT GNRs or laser alone and the experiments were repeated thrice. The parameters of GNRs and laser used in the control groups with tumor sizes measured for each mouse is given in Supplementary Tab. S5.

At specific time points for each group, fluorescence imaging was performed, followed by NIR laser irradiation of the tumor site for PTT. The result from each animal was assessed separately as a single entity. The end of study in the experimental arm was 28-30 days after the commencement of laser irradiation with an excision biopsy of the irradiated tumor site and a thorough post mortem examination of all organs.

### Experiment 1 - Administration of functionalized GNRs and non-invasive imaging

Whilst under general anesthesia (intraperitoneal ketamine and xylazine), mice received a single bolus of functionalized GNRs injected either directly into the tumor (intratumoral, IT, 50-75 μl GNRs, OD_λ = 808_ = 13.2) or systemically (intravenous via tail vein, IV, 100 μl GNRs, OD_λ = 808_ = 26.4). IT injections involved delivering approximately 12-17 μl of GNRs into each quadrant of the tumor, and not solely into its epicenter, in an endeavor to establish a homogenous spread of GNRs throughout the tumor.

**Figure 3:**
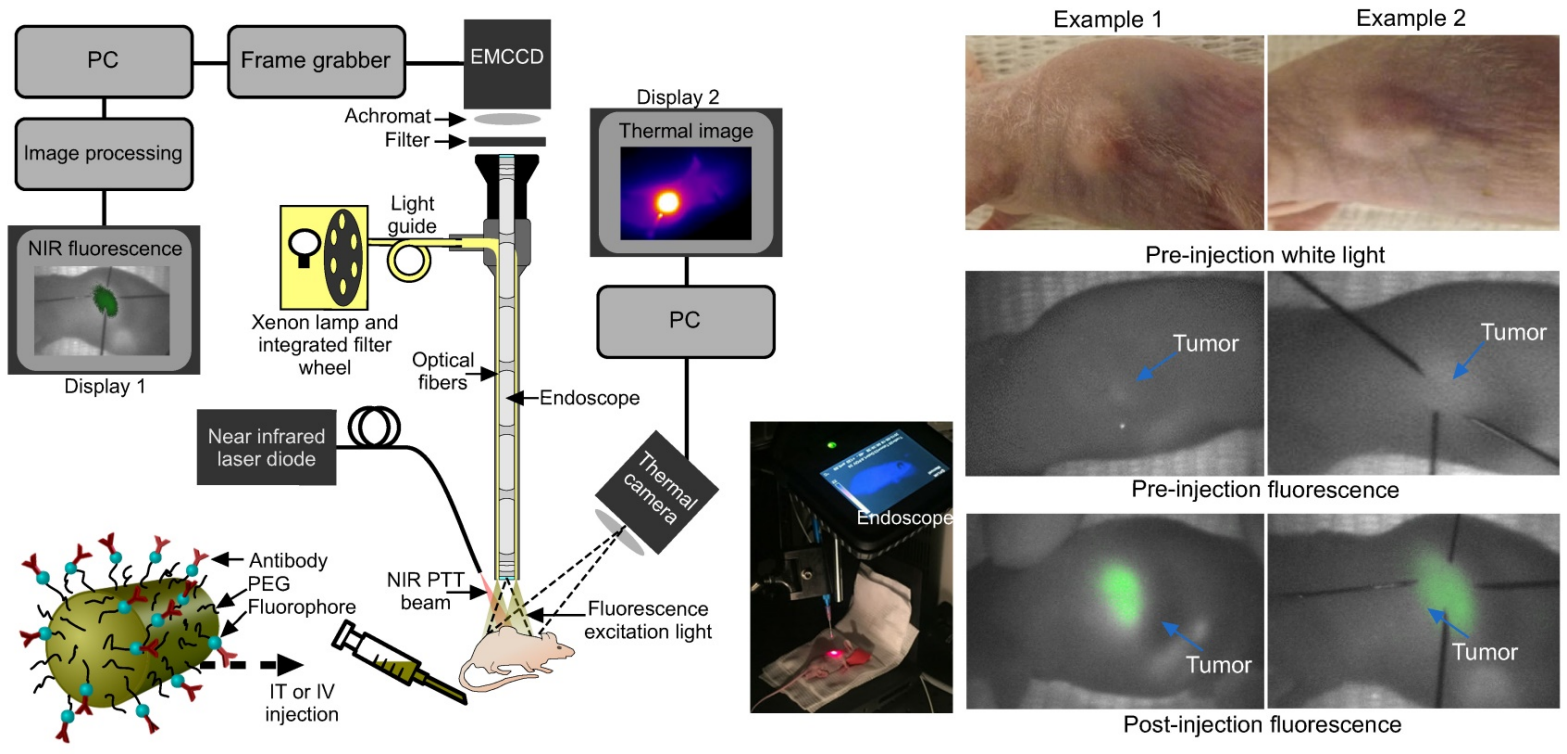
Experimental set-up and fluorescence imaging results. **(a)** Fluorescence endoscopy set-up with PTT optical fiber (~1 cm away from the specimen) and thermal imaging camera in situ, with inset photograph. **(b)** White light and endoscopic fluorescence images pre and post intravenous injection with functionalized gold nanorods for two example mice. The fluorescence emission signal arises specifically from the tumor area, as confirmed by comparison with the white light images. The post injection fluorescence image illustrates the tumor and its boundaries localized with needles for photothermal laser aim-beam alignment.

Fluorescence imaging was conducted by exciting the tumor area with filtered light from a 300 W Xenon lamp (655 nm central wavelength, 50 nm bandwidth, Omega Optical) coupled into the illumination optics of a conventional 0º laparoscope (Karl Storz, HOPKINS 870IAG). A 690 nm long pass filter (690 AGLP, Omega Optical) and 30 mm achromatic lens (Thorlabs) were inserted between the laparoscope and the camera (Hamamatsu EMCCD Camera Model C9100-13, Hamamatsu Photonics UK Limited, Hertfordshire, UK; integration times ranged from 40 ms to 5 s) in order to block the reflected light from the mouse (Fig. 3). The spatial resolution of the system comprised two factors: the image space resolution of the CCD sensor (= 31.25 line pairs/mm) and the primary magnification of the system (= 0.16). Overall the quantity of interest, i.e. the object space resolution was 100 µm. Prior to the experiments, test charts were used to determine the performance of the system at the appropriate laparoscope-tip to object distance.

Sensitivity was determined by the achievable signal-to-noise ratio (SNR). Over the wavelength range of interest, the quantum efficiency is >80%. With an integration time of 5 s and appropriate photon flux, read noise and dark current values, the SNR was ~50. Images were acquired using MatLab programming language and the images were processed and analyzed using Origin software (OriginLab Corporation). Background fluorescence was measured pre-injection, with fluorescence imaging performed again at multiple sites 0, 2 and 24 hours post injection for IV mice. The image acquisition time varied between 0.5 and 5 s, although most images were acquired in 5 s due to the higher contrast obtained. However, shorter acquisition times (<0.5 s) would have also provided enough contrast for the signal to be detected with acceptable SNR.

### Experiment 2 - Photothermal therapy and evaluation of treatment

Non-invasive near infrared (NIR) light between 1.0 and 2.6 W (maximal safe laser power for both IV and IT groups determined in pilot work) was applied for up to three minutes extracorporeally using a small, inexpensive and portable CW diode NIR 808 nm laser (DenLase-810/7 laser) aligned alongside the laparoscope. The maximal laser fluence was 5 W/cm^2^, which was ascertained from pilot work and a literature review^5^ as being the optimal laser fluence used in successful and published *in vivo* PTT experiments using gold nanoparticles. The power was selected based on the tumor size of each mouse regardless of IV or IT injection. The laser beam diameter (determined using the aim beam function of the laser) covered the tumor diameter plus an additional 1-2 mm area over the diameter of the tumor.

The combined theranostic set-up for both fluorescence imaging and PTT is shown in Fig. 3. For mice receiving IT GNRs, PTT was done within 30 minutes of delivery, while those receiving IV GNRs underwent PTT after 24 hours of delivery. This ensured there was adequate time for systemically delivered functionalized GNRs to circulate to achieve maximal intratumoral accumulation. The parameters of GNRs and laser used in the PTT groups with initial tumor sizes on each mouse are given in Supplementary Tab. S6 and S7.

### Clinical examination

Tumor sites were measured three times a week using calipers, but once treatment was instituted there were only resultant tumor eschars from photothermal therapy in mice, which were examined daily for any complications. At the end of the study period, all mice in the treatment arms of the study underwent a thorough post-mortem clinical examination of tumor sites, the peritoneum, liver, spleen, omentum and lungs at termination for an assessment of metastasis from the original implanted tumor.

### Experimental outcomes

1. Evaluate fluorescence imaging in correct identifying cancerous tissue by exploiting the physiological ability of functionalized GNPs to target tumors.

2. Confirm the ability of PTT in successfully reduce or eliminate tumors.

3. To propose a practical approach fortheranostics in clinical practice.

## Results

### Application of fluorescence endoscopic imaging for cancer theranostics *in vivo*

For mice that were subject to IV administration of GNRs, there was a good endoscopic fluorescence signal within the first hour which corresponded well to the external appearance and margins of the tumor (Fig. 3). This was highly reproducible using fluorescence imaging and could be a useful adjunct in routine endoscopy for highlighting subtle or occult tumors *in vivo* for image-guided therapy. Localization of the tumor with fluorescence imaging therefore permitted contemporaneous image-guided markings of the tumor boundaries (achieved here using hypodermic needles following fluorescence imaging). Following localization, the fluorescence excitation light was replaced with the NIR laser aim-beam, which was directed to coincide with the marked area prior to irradiation.

**Figure 4:**
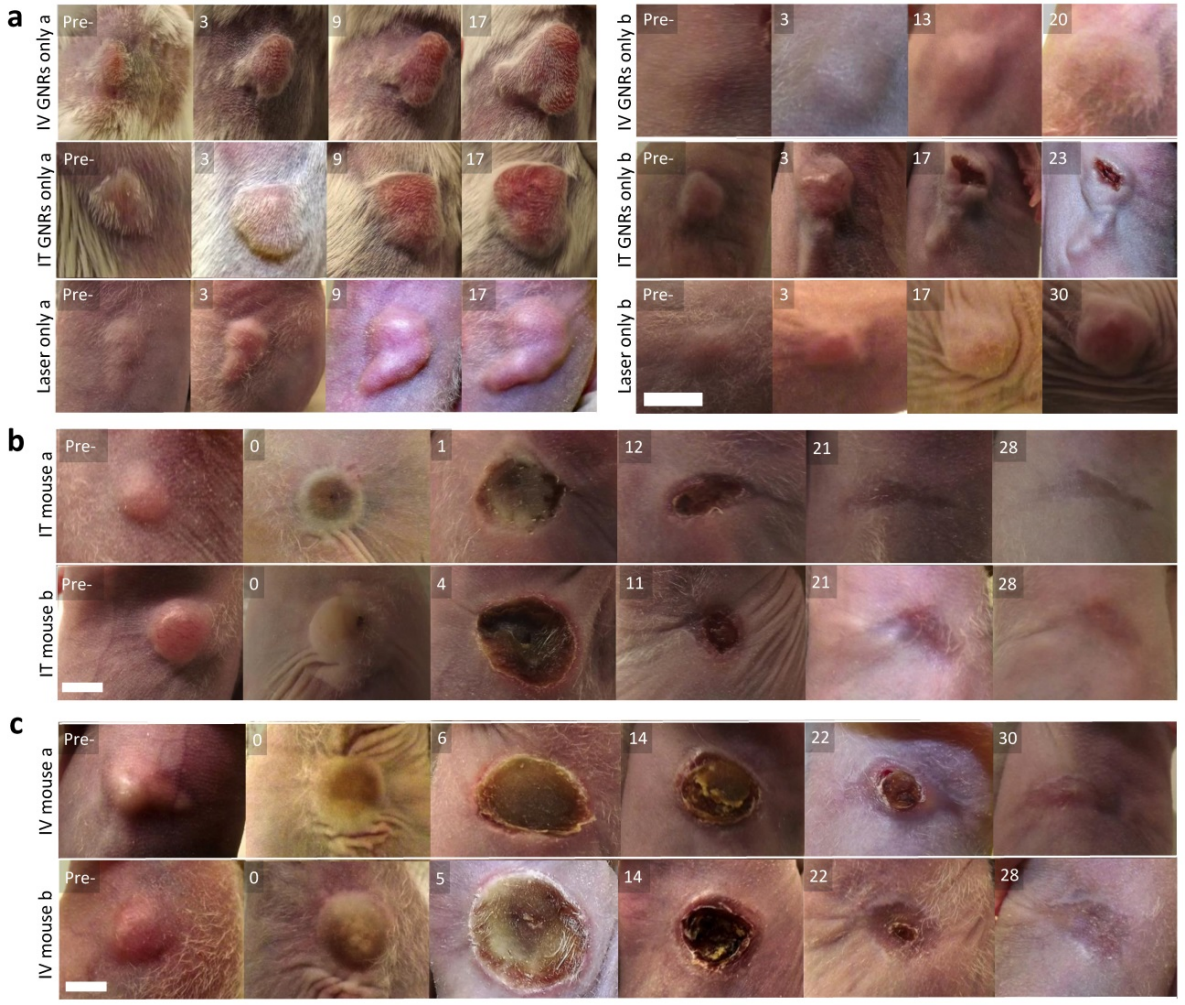
Visual appearance of tumors in mice. **(a)** Representative images within the control group, receiving either IV or IT GNRs (without laser) or laser irradiation alone (without GNRs), showing images at stated days post-intervention (Pre- is the original pre-intervened tumor), indicating continued tumor proliferation in all control arms. **(b,c)** Representative photothermal therapy responses in tumors of the 10 mice (Pre- is the original tumor pre-PTT, 0 is the tumor immediately post PTT), with images at stated days post-PTT in mice which received IT **(b)** or IV **(c)** GNRs, demonstrating complete ablation of all tumors. Photographs illustrate changes seen over days, with approximate scale bar of length 6 mm shown in the bottom left sub-image of each sub-figure. Further images are presented in Supplementary figures S2 and S3.

**Figure 5:**
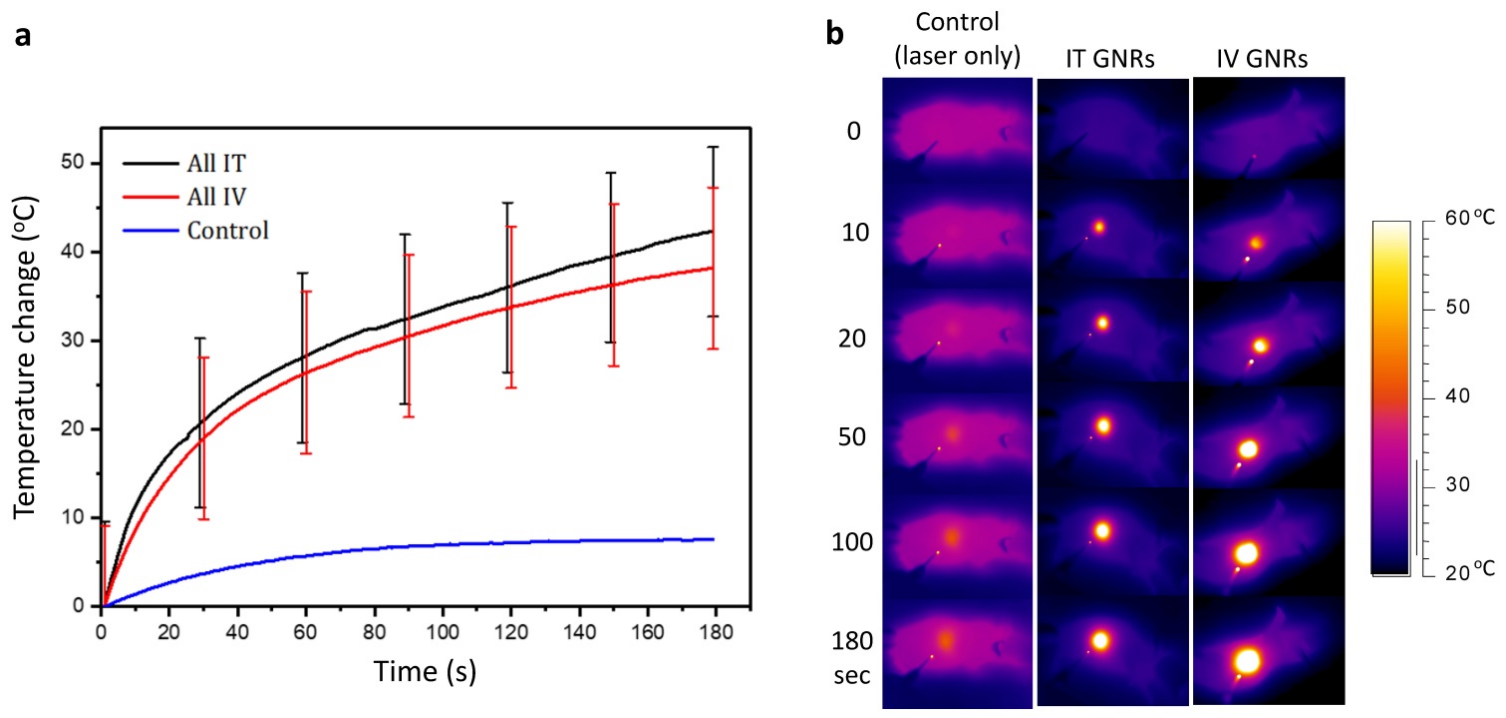
Temperature response during thermal therapy. **(a)** The mean and standard deviation of temperature rise from all mice observed during PTT in both IT and IV GNRs groups, together with a normal control. **(b)** Representative thermal camera images during PTT for the classes indicated. The background mouse temperature depended on ambient conditions and varied slightly between animals.

No tumors were found to be self-limiting and no tumor regression or dissolution response could be elicited from a single modality of either GNRs or NIR laser irradiation alone (Fig. 4a). A temperature rise within the 7.6-9.9ºC range was observed in the laser only control tumors and is likely to have been from superficial (skin surface/hair follicles) or pin-point heating, thus not generating a homogeneous heating profile throughout the entire tumor tissue. The lack of any noticeable damage to the laser-irradiated skin surface or any clinical improvement demonstrated the innocuous nature of NIR light in the absence of GNRs so long as it remained within the fluence range used. The mean temperature rise after three minutes of irradiation in the IT group was higher (42.3 ± 9.4ºC) than in the IV group (38.0 ± 9.0ºC) (Fig. 5). This is explained by the higher concentration of GNRs within the tumor site in the IT group, as subsequently objectively quantified by ICP-MS analysis (Fig. 7c). As can be seen, the temperature rise of the control mouse (laser only) is significantly lower (7.5^o^C, p<<0.01) compared to the other groups.

The temperature rise in both IT and IV groups was made to be similar by doubling the concentration, volume and optical density of GNRs given IV compared to that administered IT. This made up for the loss of GNRs in the IV group which become opsonized by the organs of the reticuloendothelial system (chiefly the liver and spleen) and the reliance on tumor targeting. The maximum safe laser power used in all the mice in our experimental studies was deemed to be 2.6 W, irrespective of the size of the tumor. It is known that much higher temperatures can be achieved with more concentrated GNRs especially when administered IT and with higher laser fluences, but this would be detrimental to the mouse, as such it was endeavored to maintain temperature rises within a certain photothermal therapy range. We note that heating is a nonlinear process due to the complex heat sinking processes, and that there is a plateau effect for the heating rates considered in this paper. When considering a human translation of this principle, the higher core human body temperature (37.2°C) would require a much lower temperature rise for intratumoral hyperthermia to be effective, and consequently a tailored approach with respect to tumor size, GNR concentration and volume, along with laser fluence and duration would need to be considered.

**Figure 6:**
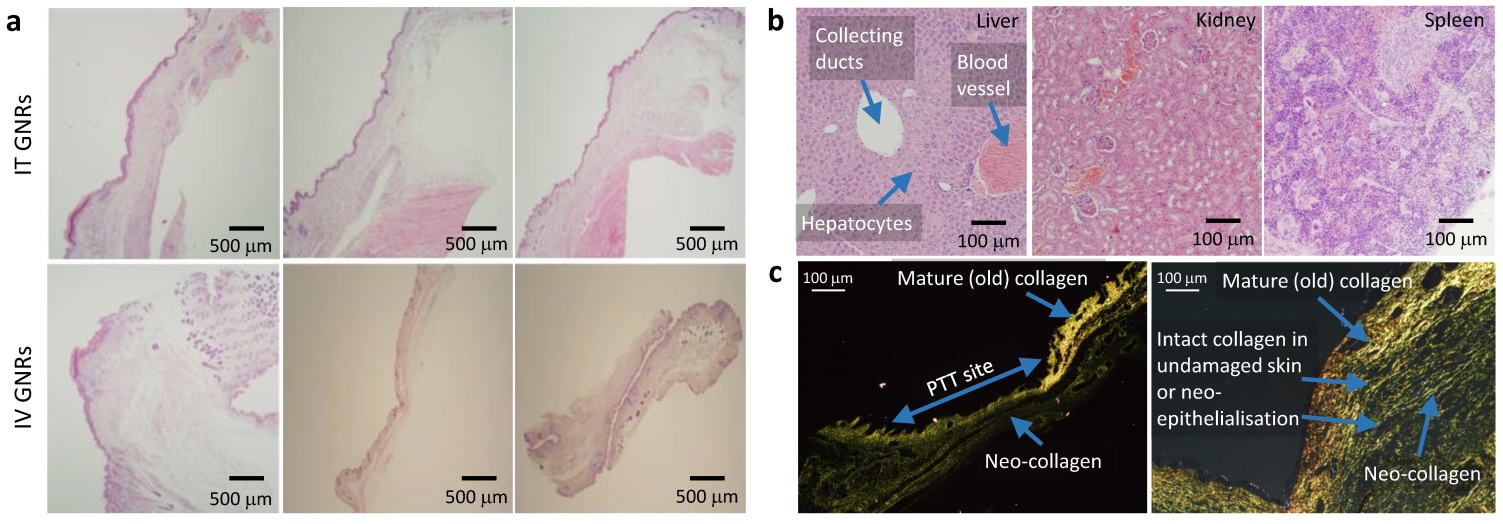
Histology of GNP distribution and PTT response. **(a)** Representative histological appearances of irradiated tumor sites in three mice following PTT in the IT and IV GNRs groups showing fully regenerated epidermal & dermal layers with no evidence of tumor or proliferating cells. **(b)** Histology from the liver, kidney and spleen 30 days post PTT showing no damage to hepatocytes, renal cells or splenocytes, showing a morphologically active spleen. **(c)** Contrast seen from re-epithelialization and regeneration of neo-collagen post PTT seen under polarized light microscopy after staining with Picrosirius red with Miller's elastic stain added showing intact collagen fibers in skin layers demonstrated following PTT.

Following tumor irradiation for PTT, there was an immediate thermal reaction observed by the crinkling/puckering of skin. Subsequently signs of tumor necrosis became evident 2-5 days post-PTT with eschar formation which would eventually be replaced by a dry scab. These scabs usually sloughed off spontaneously by the start of the third week to reveal an underlying healed irradiation site which was tumor-free by day 30 in both groups. The results from all mice in both groups demonstrated good photothermal response with complete macroscopic disappearance of tumors (Fig. 4b-c; full results in Supplementary Fig. S2 and Fig. S3) with a corresponding absence of any proliferating cancer cells on post-termination histology of the excised irradiated sites at the end of study (day 30) in Fig. 6a. Microscopically the irradiated tumor sites in both treatment arms demonstrated healthy cells with fully regenerated and well-defined layers of epidermis and dermis which had experienced neo-collagenesis, which was further confirmed under polarized light microscopy (Fig. 6c).

### Electron microscopy, mass spectrometry and clinical examinations

**Figure 7:**
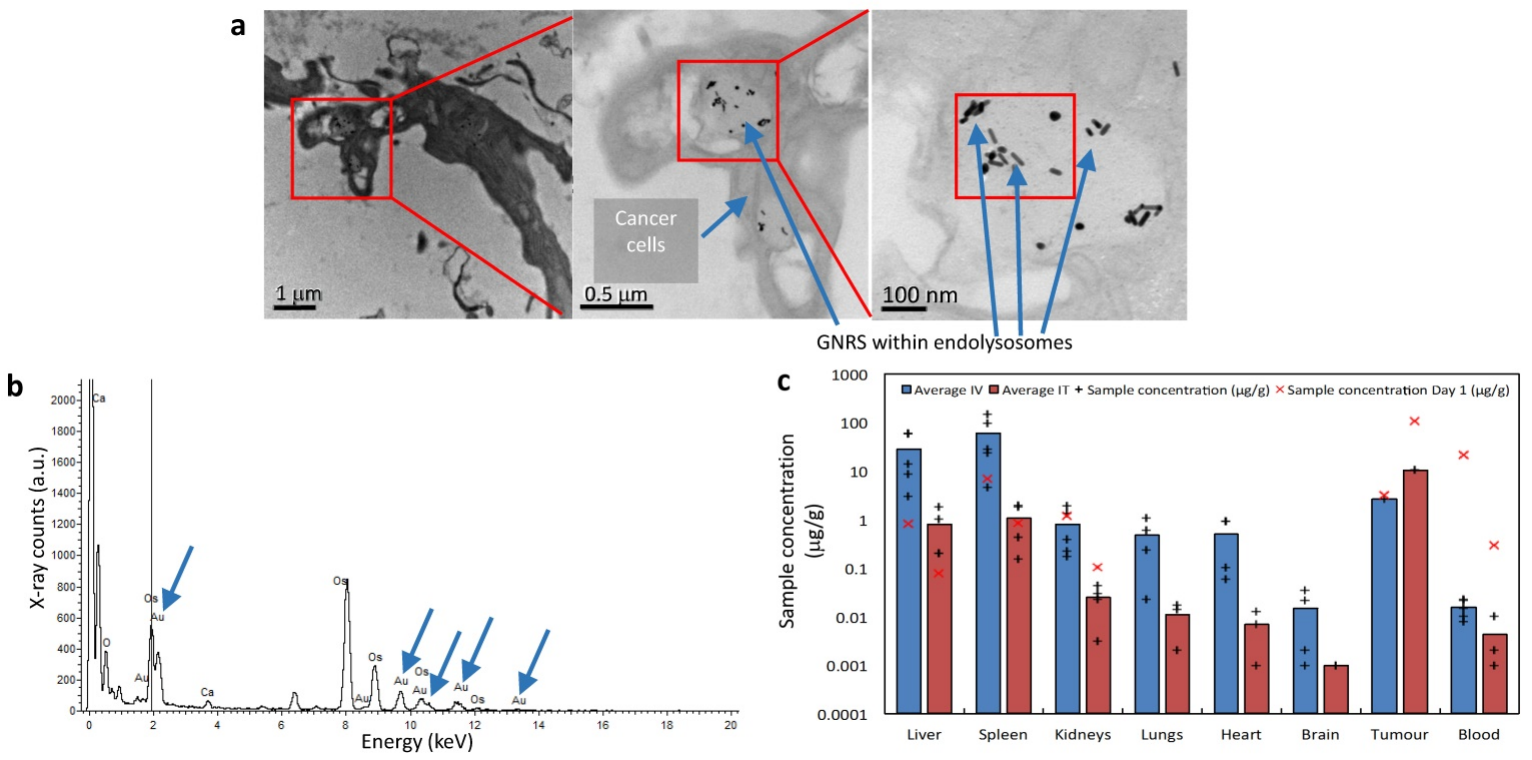
Electron microscopy and mass spectroscopy analysis of GNP distribution and PTT response. **(a)** TEM of a group of cells within the tumor tissue showing GNRs within endolysosomes. **(b)** An energy-dispersive X-ray spectrum taken from the square indicated in **(a)** showing characteristic Au peaks (blue arrows). **(c)** The average [Au] in tissues/blood at the end of study. X's correspond to [Au] at Day 0/1.

Evaluation of tumor sites of mice which received IT GNRs under TEM demonstrated that tumor tissue contained black spots (Fig. 7a). Upon increased magnification (to 120,000x), it could be resolved that these appeared to be intracellular GNRs within endolysosomes which were contained within the cytoplasm of individual cancer cells. Energy-dispersive X-ray spectroscopy (EDS/EDX) was used for further characterization of these particles (Fig. 7b). Corresponding peaks within the processed EDX analysis showed that these intracellular 'black dots' were peaks of gold (Au), which conclusively proved that these particles were indeed the administered GNRs. The other peaks observed corresponded to carbon, calcium (Ca) and osmium (OsO_4_), which are elements either found naturally intracellularly or were used as the sample fixative. Thus this provided conclusive evidence that anti-EGFR-antibody-GNRs injected intratumorally became endocytosed within cancer cells, where they were optimally poised for subsequent PTT.

The biodistribution of gold within mice was determined using inductively coupled plasma mass spectrometry (ICP-MS). Blood, tumor sites and organs such as liver, spleen, kidneys, lungs, heart and brain were harvested for analysis. The average concentrations of Au ([Au]) per organ are represented in Supplementary Tab. S1 and illustrated graphically in logarithmic scale in Fig. 7c. The average [Au] obtained via ICP-MS from various mice organs and blood at the end of study (day 30) in Fig. 7c was measured and contrasted with values obtained from mice receiving only GNRs at Day 0 (akin to control mice). Day 0 values correspond to post-GNR administration values and were included to give a representation of what the [Au] was at the point at which PTT would be instituted, however irradiation was instead withheld and the organs, blood and tumor sites were harvested. The tumor sites harvested had similarly not been exposed to radiation, and the [Au] was measured at days 0 and 17, which was the end point due to gradual increase in the size of tumor without PTT.

Intravenously administered GNRs resulted in a higher accumulation of Au in all studied organs compared to IT delivery, except within the tumor itself. The mean temperature rise following irradiation in the IT group was higher than the IV group due to the higher concentration of GNRs within the tumor site in the IT group. ICP-MS revealed that a proportionately larger volume and optical density of GNRs was required intravenously to achieve similar temperature rises and tumor ablations to IT injections because more GNRs were being sequestered by the liver and spleen when administered intravenously, and consequently a reduced tumor concentration of GNRs. The brain, lungs, heart and kidneys showed virtually negligible accumulation of Au in comparison to the proportionally higher accumulation in the organs of the reticuloendothelial system (RES), chiefly the spleen and liver, which are known to be the primary organs of GNP biodistribution and metabolism^27^-^37^. Fig. 7c suggests there is a gradual accumulation of Au particles within the liver and spleen from the day of administration to the end of study, which is counterpoised by the gradual ebb from blood and tumor sites. The higher [Au] seen in the spleen may also be partly due to the relatively smaller size of the spleen in comparison to the liver, thus accounting for more Au per gram of organ. Histological examination of the liver, spleen and kidneys of mice by light microscopy at the end of the 30-day study period demonstrated that there was no structural damage to these organs or their respective cells (Fig. 6b), despite there being a trace of Au within these organs.

The significance of conjugating a targeting agent such as anti-EGFR-antibody onto GNRs becomes relevant during systemic (IV) administration due to sequestration of a large proportion of GNRs within reticuloendothelial organs. In our pilot studies using non-functionalized IV GNRs, tumors in mice were neither fluorescent under endoscopy (due to the lack of a fluorescent dye) nor adequately ablated when relying solely on a passive EPR effect of tumor targeting as they were with antibody-functionalized GNRs. The data of [Au] from ICP-MS within tumors provided conclusive evidence however that the multifunctionalized GNRs were arriving at tumor sites from systemic (IV) administration. This confirmed the superiority and value in fabricating multifunctional GNRs as they were clearly reaching their target destination through a combination of active and passive processes, by demonstrating their effective theranostic effects. The [Au] within the circulating blood volume showed a negligible quantity of Au from both methods of administration at the end of the study (30 days). Clearance of Au from the bloodstream is important in ensuring there would be no long-term consequences from systemic reservoirs of GNPs which may aggregate/coalesce and propagate as emboli. All mice were autopsied at the end of the study and a thorough inspection of the peritoneum, liver, spleen, omentum and lungs indicated that there were no detectable metastases from the original implanted and ablated tumor.

### Blood, morbidity, physical and psychosocial attributes

Blood tests carried out on mice receiving PTT with both IV and IT routes of administration did not demonstrate any hematological or biochemical deviations from normal parameters occurring as a consequence of GNRs or PTT at the end of the study (see Supplementary Tab. S2). It can therefore be inferred that the intrinsic function of livers and kidneys is preserved.

No mouse in either treatment arm lost weight from the start of inoculation to the end of study (see Supplementary Fig. S4, Supplementary Tab. S3). No mouse demonstrated muscle atrophy or emaciation, and no animal experienced anorexia (loss of appetite) or appeared dehydrated at any point during the study. In our study no systemic side effects or morbidity were observed during the study period and there was no unexpected mortality. Once awake from general anaesthesia, it was apparent that the animals did not experience undue pain as a consequence of PTT, such that no animal appeared to require analgesia before or after PTT. The sites of tumor inoculation and PTT were consistently dry and clean. There was no observable change in the physical or psychosocial attributes of mice receiving GNRs alone or with PTT, even when administered at the higher (1.5x or double the normal) IV dose performed in pilot studies.

## Discussion

Theranostics refers to agents that are simultaneously therapeutic and diagnostic. Theranostics using GNPs utilizes an efficient system to diagnose and deliver targeted therapy. When excited with a wavelength of light attuned to the specific surface plasmon resonance (SPR) of GNPs, surface electrons on GNPs develop very strong oscillatory energy, which in turn generates high temperatures which cause localized tissue death^5^. When GNPs are heated within cancer tissue, the resultant photothermal reaction can be used to destroy cancer cells within tumors (PTT). To harness the *in vivo* potential of applying this photothermal effect to cancer tissue, the SPR of GNPs is tuned to absorb NIR light^5^ by manipulating their aspect ratio (length/width) during chemical fabrication. The clinical superiority of NIR light resides in its minimal attenuation by water, hemoglobin and endogenous chromophores, such that it can penetrate soft tissues at depths of up to 10 cm^38^. The application of gold nanospheres have rather limited spectral tunability due to their resonance peak at ~ 520 nm in the visible, which considerably limits their clinical application in GI cancer due to the absorption and scattering of this light by tissue and chromophores^5^.

Both IV and IT GNR administration routes achieved complete clinical remission of tumors by the end of the study period, as confirmed by conventional histopathology. The tumors were of an appropriate size (5 mm), balanced to be both small enough to be useful in detecting small lesions yet sizable enough to be clinically relevant when ablated. It is clear from the control studies that when not successfully treated by PTT, tumors would continue to grow exponentially large. IV GNRs are clinically applicable in aiding the diagnosis of subtle or occult cancers via fluorescence endoscopy, while IT GNRs remains a feasible solution to overcome the issue of molecular heterogeneity within tumors and is therefore clinically relevant for therapeutic intervention.

This work has shown effective use of multifunctional GNRs in the theranostics of upper GI cancer and formulated a novel and practical design for real-time *in vivo* application via endoscopy. We have shown that the incidence of missed cancers during endoscopy may be mitigated by enhancing the optical contrast afforded by fluorescence endoscopy attained through highly selective nanoparticle targeting. Submucosal esophageal and gastric cancers can be hidden from the naked eye during white light endoscopy and thus be missed due to the normal overlying mucosa. In our experiments, despite a healthy overlying skin, the underlying subcutaneous tumors did not escape GNR-induced fluorescence illumination and detection. It is plausible to transfer this set-up to a clinical endoscopic setting, with fluorescence excitation light replacing standard Xenon white light, where large areas such as the entire mucosal surface of the stomach or peritoneal cavity can be visualized live under fluorescence imaging during endoscopy/laparoscopy. This can support *in situ* detection of early or occult cancers through optical enhancement by live fluorescence imaging, which can in turn guide optical biopsies and targeting for photothermal therapy. For intracorporeal use, NIR fiber optics can be introduced into the working channel of any commercially available flexible endoscope or through any 5 mm laparoscopic port during minimally invasive surgery. For contemporaneous tumor ablation, the laser beam can be delivered directly to the tumor target area which would be clearly defined by the illumination afforded by fluorescence imaging.

Anti-EGFR antibody was the ligand of choice on GNRs as EGFR is not only overexpressed in esophageal cancers^24^, but numerous other cancers^39^ and hence the results shown here could potentially be transferable to other cancers. Although competitive binding is expected with other EFGR-expressing organs, targeting of functionalized GNRs to tumor sites using the synergistic combination of active targeting (antibody-driven) and passive accumulation (EPR effect) amplified GNRs within the tumor matrix to enhance effective PTT. With mass spectrometry and microscopy, it was demonstrated that despite some accumulation of GNRs within the liver (whether by GNR metabolism or competitive EGFR expression), there was no effect on hepatocytes, however the effect on tumors *in vivo* was substantial and clinically relevant. Within the tumor sizes in this study, therapy seemed to be effective with just a single application of GNRs and a short irradiation period with relatively low energy. An aggressive human esophageal adenocarcinoma was selected as the tumor model in this *in vivo* study as it is representative of other solid cancers and thus the clinical significance and translation of this technology to other cancers is envisaged.

Controversy exists regarding the superiority of PTT over photodynamic therapy (PDT) with regards to their respective efficacies in ablating esophageal tumor in several *in vitro* as well as pre-clinical *in vivo* experiments. PDT employs organic photosensitizers, which, when excited by a specific wavelength of light, produce (singlet) oxygen radicals capable of destroying nearby cells. GNRs have the distinct advantage of being able to provide optical contrast and may additionally be functionalized with chemotherapy pro-drugs that become activated within cancer sites. Unlike PDT, PTT does not require oxygen radicals to interact with the target cells or tissues. Current studies show that PTT is able to use more stable and longer wavelength light (NIR light), which is less detrimental to other cells and tissues, and may penetrate deeper into tissues than the light used for PDT (UV and visible light)^40^. Hence conventional PDT is limited to surface tumors, and not applicable for deeper tumors. Conventional organic photosensitizers used in PDT also suffer from severe photobleaching and enzyme degradation. While PTT specifically acts on cancer cells, photosensitizers used in PDT are not specific because they are also absorbed by ambient healthy tissues and can induce burns, pain, swelling and intense fibrosis within healthy tissues. When assessing suitability for clinical efficacy and therapy, a nanovector should fulfill the criteria of (a) NIR light activation for deep tissue penetration; (b) resistance to photobleaching; (c) enzyme degradation; and (d) high extinction coefficients for effective light absorption^41^. GNRs fulfill all these criteria by being resistant to photobleaching, enzymatic degradation, and by possessing a 4-6 orders higher extinction coefficient which permits more effective light absorption than common photosensitizers^41^. Hence in this regard GNRs are significantly more effective than photosensitizers as multifunctional theranostic agents.

The therapeutic potential offered by functionalized GNRs may have applications in both the early and late stages of cancer. In early gastrointestinal cancers (T1-T2) that are either inappropriate or unfavorable for radical surgery or have failed attempts at endoscopic mucosal resection or endoscopic submucosal dissection, PTT may offer an alternative modality of treatment. For advanced cancers, thermal ablation such as that used for resectable colorectal liver metastases is seen as a viable alternative to surgery (hepatectomy) such that a new multicenter randomized control trial opened in 2016 in the UK comparing disease free survival at 2 years from both treatment arms^42^. Other potential applications of photothermal therapy would be in isolated lung or peritoneal metastatic lesions, tumor ingrowth within intraluminal stents, large polypoid lesions and malignant melanoma. It is further envisioned that PTT could be directed to tumor cavities post-operatively, or be applied in margin positive or partial resections. By delivering a high tumor concentration of GNRs, endoscopic IT injection of GNRs followed by PTT could induce effective tumor debulking. Treatment can be staged and repeated, thus applicable in the palliation of symptoms arising from inoperable GI and hepatic tumors.

While this *in vivo* study used a subcutaneous tumor xenograft model, it would be useful to know if the results seen with fluorescence imaging and PTT are also effective for orthotopic tumors, and can afford similar optical contrast enhancement from intraluminal tumors. Orthotopic evaluation of *in situ* irradiated tumors would permit an assessment of any complications arising from *in situ* PTT, such as perforation or bleeding, apart from also being able to quantify the safe levels of GNRs and laser power that can be utilized. To validate this further, it would be prudent to commence with a short laser exposure time and increase according to dose effect. Although orthotopic tumors are ideal in evaluating clinical application and translation, it was not plausible to perform fluorescence imaging combined with photothermal energy via an exceptionally small gastroscope in our mouse model which also relied heavily on an immunodeficient status and sterile environment in order to establish and maintain solid human adenocarcinoma tumors, and the technology would also not be directly translatable to human scale. Larger animals were considered for endoscopic experiments on orthotopic tumors, however the yield of generating and sustaining reliable gastrointestinal cancers on large animals is significantly dismal.

Future work may focus on determining the optimal time for imaging and therapy, which would involve a more extensive study. This may involve performing regular interval fluorescence intensity quantification, as well as PTT at similar time points. An alternative would be to perform biopsies of tumors at various time points and establish the [Au] within the tissues. While it is known that there were no 30 day effects seen histologically within hepatocytes and splenocytes, and mice did not experience any apparent physical, physiological or psychosocial deterioration, it would be noteworthy to ensure this could be sustained over a longer period, and repeat biodistribution profiling at lengthier timepoints.

When evaluating the safety of *in vivo* GNRs and PTT as analyzed from the multi-source results from histology, mass spectrometry, post mortem evaluations, clinical examinations, psychosocial behavior evaluation, blood tests and weight monitoring, it has by all accounts, provided a robust safety profile of all the individual components. This strongly suggests that when viewed as a whole, the application of GNRs as a theranostic tool in conjunction with endoscopy is potentially safe and effective for GI cancer detection and therapy. The proposed image-guided therapy that ensures complete ablation of cancer with minimal collateral damage has the potential to overcome the inherent challenges of incomplete resection encountered with current endoscopic techniques.

## Conclusion

The theranostic potential of gold nanorods and its practical clinical application via endoscopy and image guided therapy have been described. The delivery of multifunctionalized GNRs in conjunction with fluorescence endoscopy has demonstrated considerable optical enhancement of tumors. Consistently successful ablative results seen from synchronous photothermal therapy of tumors were achieved using a single application of GNRs and a short irradiation period with relatively low energy.

The integration of nanotechnology with endoscopy for imaging and targeted treatment with effective and reproducible results could reduce the incidence of missed early lesions, maximize tumor cell death while minimizing damage to healthy tissues. Being shown to be a viable and safe adjunct to endoscopy as a minimally invasive procedure, the clinical application of photothermal therapy will allow precise curative treatment and reduce potential complications.

## Supplementary Material

Supplementary methods, figures and tables.

## Abbreviations

CT: computed tomography
CTAB: cetyl trimethylammonium bromide
Cy5.5: cyanine5.5
DMEM: Dulbecco's modified Eagle medium
EDC/NHS: N-(3-Dimethylaminopropyl)-N′-ethylcarbodiimide hydrochloride/N-Hydroxysuccinimide
EDS/EDX: Energy-dispersive X-ray spectroscopy
EGFR: epidermal growth factor receptor
EGJ: esophago-gastric junctional
EMCCD: electron multiplying charge coupled device
EPR: enhanced permeability and retention
GI: gastrointestinal
GNP: gold nanoparticle
GNR: gold nanorod
ICP-MS: inductively coupled plasma mass spectrometry
IHC: immunohistochemistry
IT: intratumoral
IV: intravenous
MRI: magnetic resonance imaging
NIR: near infrared
OCT: optical coherence tomography
PEG: polyethylene glycol
PET: positron emission tomography
PPL: project license holder
PTT: photothermal therapy
RES: reticulo-endothelial system
SCID: severe combined immunodeficiency
SPECT: single photon emission computed tomography
TEM: transmission electron microscopy.
