# Supplementary material for: Laparoscopic fluorescence image-guided photothermal therapy enhances cancer diagnosis and treatment
Source: Nanotheranostics. 2019 Jan 29;3(1):89–102. doi: 10.7150/ntno.28585 (PMC6427937; doi:10.7150/ntno.28585)
Supplement: Supplementary file 1 — Supplementary methods, figures and tables. [file ntnov03p0089s1.pdf]

Supplementary methods section

Materials for GNR synthesis and functionalization

CTAB was purchased from Calbiochem, while silver nitrate ($AgNO_3$), gold (III) chloride trihydrate ($HAuCl_4 \cdot 3H_2O$), sodium borohydride ($NaBH_4$), L-ascorbic acid (AA), N-(3-Dimethylaminopropyl)-N'-ethylcarbodiimide hydrochloride (EDC), N-Hydroxysuccinimide (NHS) and Potassium Carbonate (K_2CO_3) were all purchased from Sigma Aldrich. Heterobifunctional thiol carboxyl PEG ($HS\text{-}(O\text{-}CH_2\text{-}CH_2)_n\text{-}(CH_2)_2\text{-}COOH$, MW = 2 kDa) was purchased from Nanocs, while DNAse/RNAse-free distilled water and phosphate buffered saline (PBS) solution were obtained from Life Technologies. Purified water (18.2 MΩ· cm) was produced by a Millipore water purification system. PerCP/Cy5.5 anti-human EGFR antibody was purchased from BioLegend® UK. Refer to Supplementary figure 1 for an overview of the synthesis process.

Preparation of the seed solution

9.75 ml of 0.1 M CTAB solution was added in a glass vial at 30°C under slow stirring. Meanwhile, 10 ml of 0.01 M $NaBH_4$ solution was prepared and inserted in ice for at least ten minutes. 0.25 ml of $HAuCl_4$ solution (0.01 M) was added into the CTAB solution followed by the addition of 0.6 ml of $NaBH_4$ solution under vigorous stirring. During this time, the color of the solution should immediately change from bright yellow to brown. After five minutes, the solution was left undisturbed for one hour.

Growth of gold nanorods

The seed solution was then used to make GNRs. Different amounts of 0.01 M $AgNO_3$ (17 mg in 10 ml) in a water solution (130 µl $AgNO_3$ to achieve a GNR aspect ratio of 3.9, 0.01 M) were added to 9.5 ml of CTAB solution (0.1 M) under slow stirring at 30°C. Then 0.5 ml of $HAuCl_4$ solution (0.01 M), 55 µl of 0.1 M ascorbic acid (AA) solution and finally 12 µl of seed solution were also added to the mixture at 30°C. The color of the solution changed from bright yellow to colorless after AA was added. The mixture was stirred for one minute and then left undisturbed in the dark for one hour. The color change noted at approximately 15 minutes indicated the successful synthesis of GNRs.

After an hour, the solution was transferred into an Eppendorf tube and centrifuged at 8500 rpm for 15 minutes at 30°C. Then the supernatant was decanted and the pellet was resuspended in 10 ml of phosphate buffered saline (PBS) (pH = 7.4). The GNR solution was stored at room temperature.

PEGylation of GNRs

Briefly, 10 ml of synthesized GNR solution was centrifuged twice at 8500 rpm for 15 minutes and then resuspended in 4 ml of water in order to reduce the CTAB concentration. Subsequently, 2 ml of K_2CO_3 buffer (2 mM) and 2 ml of HS-PEG-COOH solution (maintaining the stoichiometric ratio between GNRs and HS-PEG-COOH at 500,000:1) were added. The solution was sonicated for two hours to prevent aggregation and then left under continuous agitation overnight at room temperature in the dark. Next, the solution was centrifuged at 8500 rpm for 15 minutes at 30°C. The supernatant was decanted and the pellet precipitate was redispersed in 10 ml of PBS buffer (pH = 7.4) in an Eppendorf tube covered with aluminum foil. Spectral and ζ-potential measurements were performed before and after ligand conjugation to confirm functionalization of GNRs with thiol carboxyl PEG (COOH-PEG-GNRs).

Functionalization of GNRs

In a classical experiment, 4 ml of GNR-PEG-COOH solution was placed into a glass vial. Then 0.5 ml 0.4 M 1-Ethyl-3-[3-dimethylaminopropyl] carbodiimide hydrochloride (EDC) and 0.5 ml 0.1 M N-hydroxysulfosuccinimide (NHS) agents were added respectively. The mixture was put on a rotary shaker for 20 minutes and then centrifuged at 8500 rpm for 15 minutes. The supernatant was decanted and distilled DNAse/RNAse-free water was added to the precipitate to make up to a total volume of 4 ml before adding modified Cy5.5 anti-human EGFR antibody (GNR:Anti-EGFR = 1:1000; PerCP/Cy5.5 anti-human EGFR antibody, BioLegend®, London, UK). The solution was left on a rotary shaker for four hours and then centrifuged. The supernatant was decanted and the pellet was redispersed in 2 ml of distilled DNAse/RNAse-free water in an Eppendorf tube covered with aluminum foil. Absorption spectra, ζ-potential measurements and fluorescence emission spectra were checked to verify GNR-PEG-Anti-EGFR-Cy5.5 conjugation (see Supplementary Fig. S5). The final solution was stored in the fridge at 4°C.

Heat profile assessment
Background and baseline body temperatures were pre-determined prior to laser application using a thermal imaging camera (FLIR Systems ThemaCAM S65 Infrared Camera – 76,000 pixel display, Wilsonville USA) mounted above the specimen. This was manually triggered at the beginning of the irradiation and set to acquire images at 1 Hz frame rate for the length of the irradiation. The CW laser was also manually triggered at the start of the thermal image acquisitions. The heating effect on both healthy and tumor tissues were observed. Temperature rises were calculated and plotted by measuring the heating occurring in a manually selected region of interest (ROI) within the irradiated tumor site and subsequently subtracting from the background temperature which was defined as an area remote to the mouse.

Psychosocial, weight and tumor site assessments
Animals were observed for four weeks during which serial photographs of progress were taken. The animals were weighed once weekly while tumor and/or irradiation sites and psychosocial behavior was reviewed daily. Monitoring was by at least thrice weekly clinical examinations and physical measurements of tumor size if there was still a tumor present post irradiation.

Histological assessment
Tissue from irradiated tumor sites was excised for microscopic assessment in all mice at the end of the study period. In selected cases histological examination was conducted on major organs. The specimen was fixed in 10% neutral buffered formal saline and embedded in paraffin prior to performing 5 μm sections which were then stained with hematoxylin & eosin (H & E) and 0.1% Picrosirius red with Miller's elastic stain to demonstrate collagen. Histological slides were imaged with an Olympus BX 40 light microscope and with polarized light microscopy.

Transmission electron microscopy (TEM) and EDX analysis
Tissue from tumor sites was harvested during terminations in mice that received either IV or IT GNRs (without laser) and also from tumor sites that underwent PTT. This excision was done to visualize the position of GNRs within the tumor tissue from both routes of GNR administration, both before and after irradiation. The tissues were visualized by TEM (TEM JEOL JEM-2100FX, Tokyo, Japan, operated at 200 kV). Energy-dispersive X-ray spectroscopy (EDX/EDS, X-Max 80 mm^2, Oxford Instruments, UK) was used to detect the presence of Au (gold) within tissues. A minimum of 20 cells per region of interest (ROI) were observed from each tissue (n=2), with a total of 40 cells viewed.

Tissues for TEM were processed as follows: Briefly, dissected tissue specimens were washed in 0.9% NaCl (saline) solution followed by another wash with sodium cacodylate buffer solution (pH = 7.4), before they were fixed with 2.5% glutaraldehyde solution at 4°C for two hours. The tissue specimens were then washed three times with sodium cacodylate buffer solution, and stained with 1% osmium containing 2% of potassium ferrocyanide for two hours and washed with distilled deionized water (x3) and incubated overnight with 1% uranyl acetate in cacodylate buffer at 4°C before *en bloc* Walton's lead aspartate staining was performed. The samples were subsequently dehydrated with 25%, 50%, 70%, 90% and 100% anhydrous ethanol for 15 minutes (x3) each, before being placed in anhydrous ice-cold acetone and left at room temperature for 20 minutes (x3). Durcupan ACM was used as the resin. The samples were then placed into 25%, 50% and finally 75% Durcupan:acetone for two hours each. Tissues were placed in 100% Durcupan overnight then into fresh 100% Durcupan for two hours. The resin block was then precision sectioned with a diamond knife ultramicrotome. For TEM analysis, sections of fixed and embedded cells were cut with an ultramicrotome using a 35° wedge angle diamond knife and floated on distilled water. Sections were immediately collected on uncoated 300 mesh copper grids (Agar Scientific, Stansted, UK) and dried for 30 minutes at 37°C. Sections of 70 – 100 nm thickness were used for imaging studies.

Blood profile assessment
Under terminal general anesthesia blood was drawn by intracardiac venesection for clinical pathology screen from mice receiving both IV and IT GNRs and PTT at the end of 30 days. Blood was examined for biochemical parameters which included urea, creatinine, liver function and a full blood count. These blood values were compared to published normal parameters for the type of mice and used to identify any systemic toxicity, hematological or biochemical abnormalities from either GNRs or PTT (results shown in Supplementary Tab. S2).

Inductively coupled plasma mass spectrometry (ICP-MS)
The concentration of gold ([Au]) within organs was determined at the end of the study using ICP-MS. This was performed on blood using intracardiac venesection during terminal anesthesia, and harvesting organs such as liver, spleen, kidneys, lungs, heart, brain and tumor sites. The [Au] at 30 days (or end of study) was also contrasted with [Au] at the tumor sites and in vital organs on Day 0 (defined as the day after IV injections of GNR or the day of IT injections) to see the initial [Au] concentration values at the time when PTT would be instituted and to observe its variation at the end of 30 days.

Tissue samples were placed in individual microwave digestion vessels with 7 ml concentrated (15.4 M) HNO_3 and 3 ml 30% H_2O_2 (Suprapur 30% H_2O_2 from Merck) and left overnight at room temperature. Blood samples were weighed into the microwave digestion vessels. Sample solutions were produced using an Ethos EZ microwave digestion system, whereby the samples were increased up to a temperature of 210°C over one hour. Once clear sample solutions were obtained, they were evaporated to dryness before dissolving in 5% aqua regia. Samples were analyzed for the gold concentration using an Agilent 7700x ICP-MS. The 15.4 M HNO_3 and 12 M HCl used in the aqua regia were purified in-house using sub-boiling distillation with quartz stills.

Supplementary figures

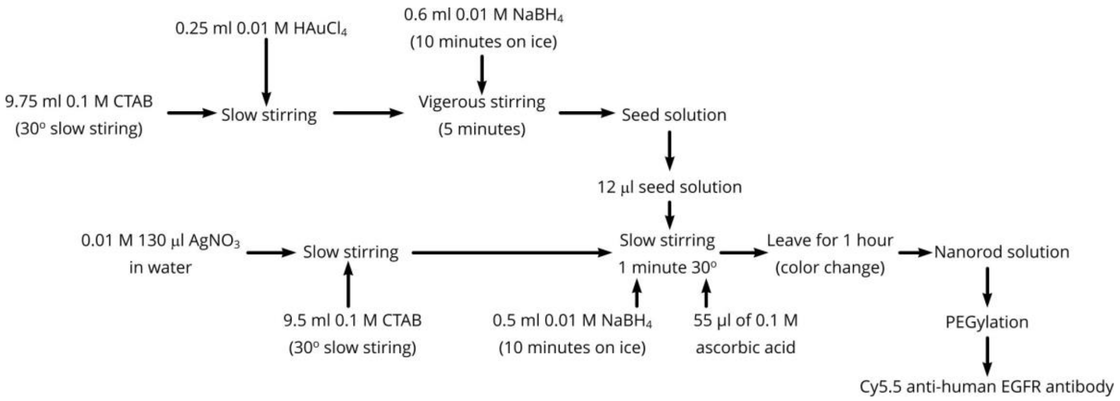

Supplementary figure S1. Summary flow diagram of nanorod synthesis and functionalization.

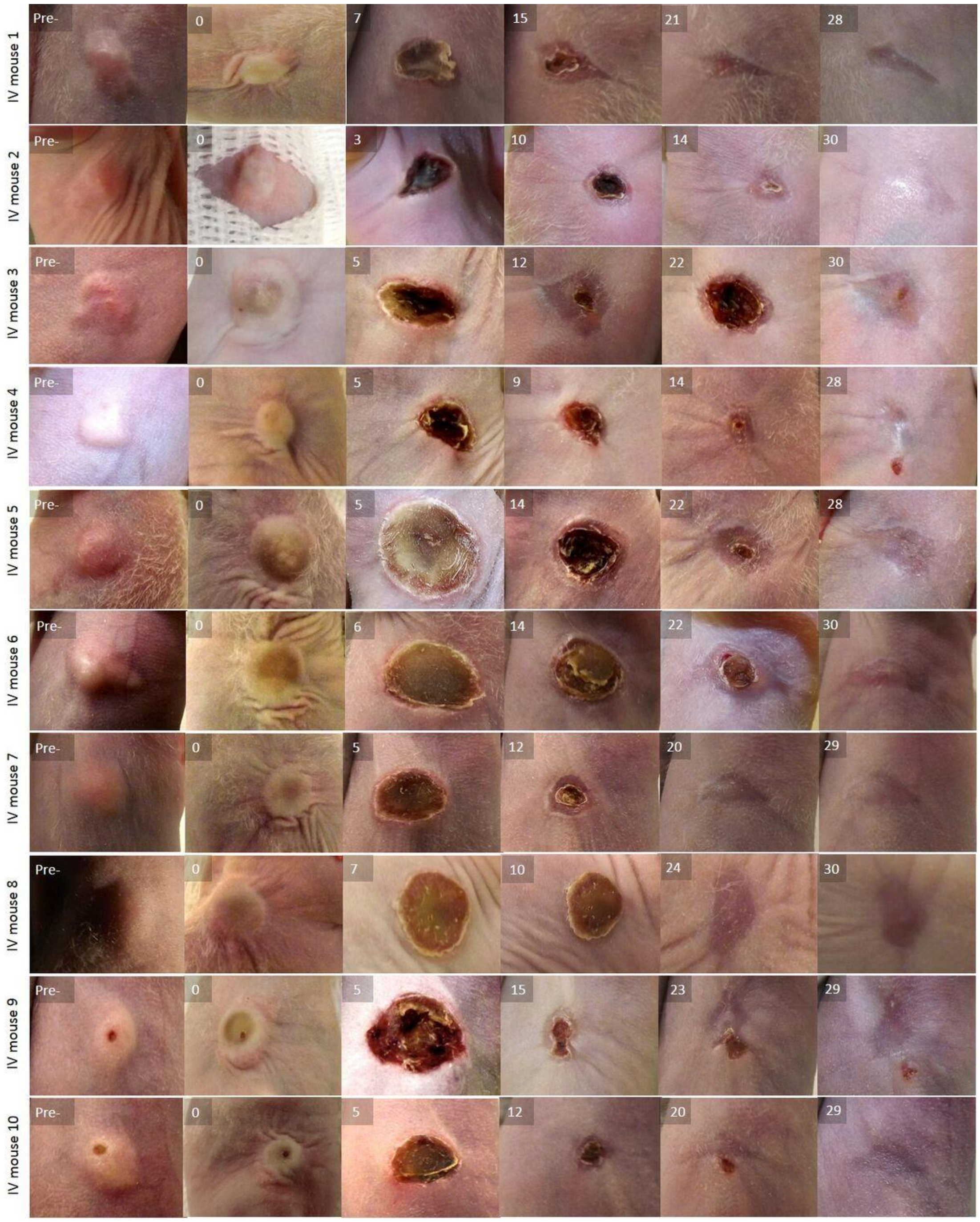

Supplementary figure S2. The PTT responses in tumors of the ten mice (initial tumors on the left column) which received IV GNRs, with their progression over days.

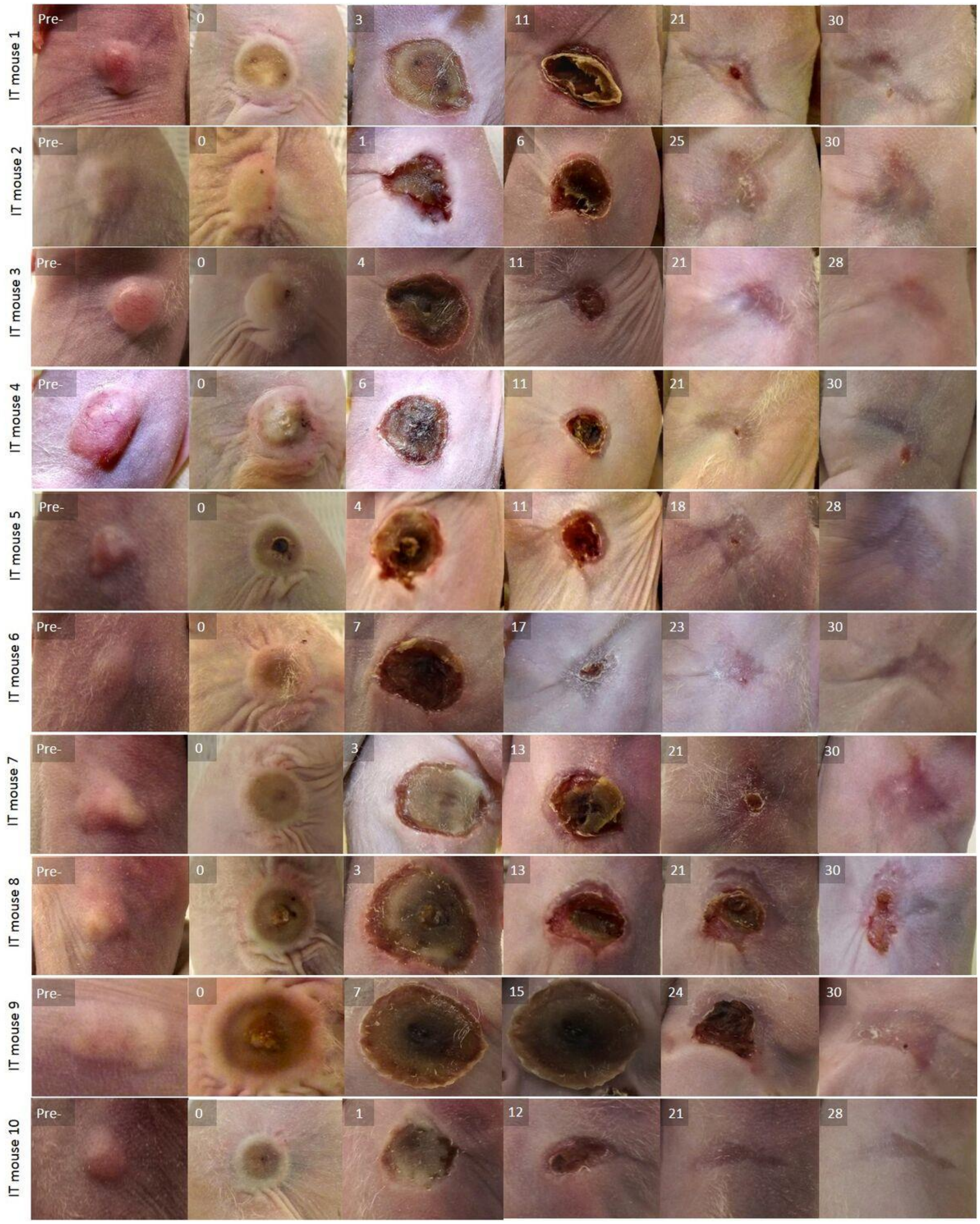

Supplementary figure S3. The PTT responses in tumors of the ten mice (initial tumors on the left column) which received IT GNRs, with their progression over days.

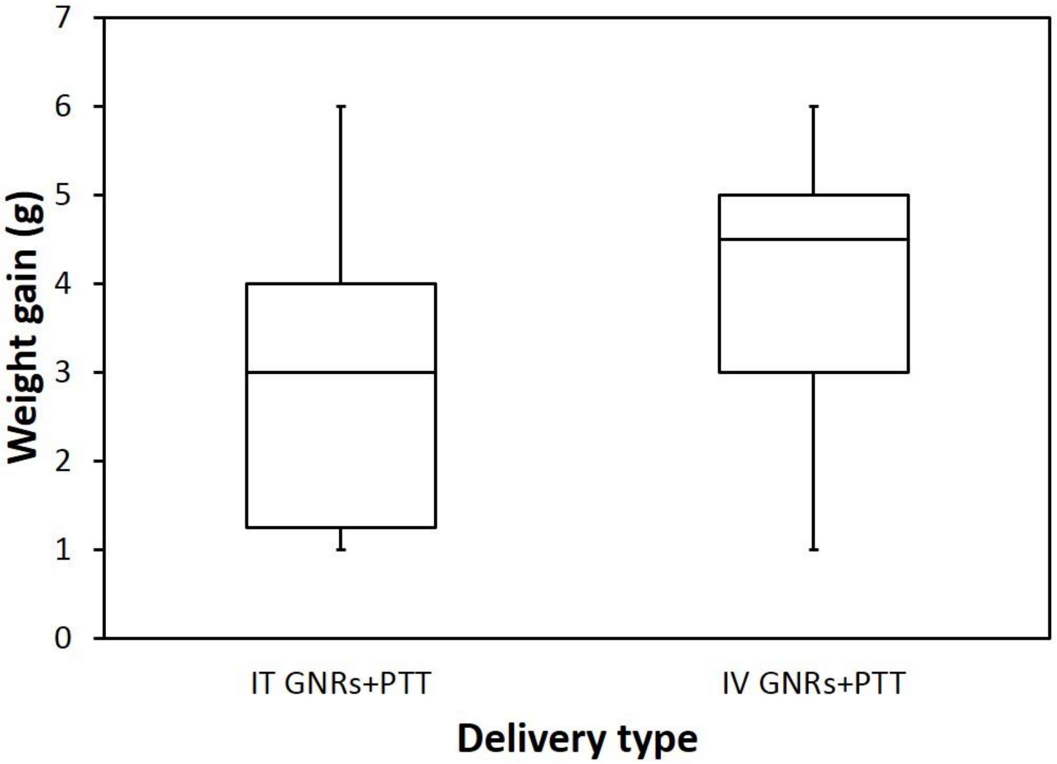

Supplementary Figure S4. Mice weight distribution. Box and whisker diagram of weight distribution of the mice, demonstrating an increase in both groups with the medians, quartiles and minimum and maximum weight gains.

Supplementary tables

Supplementary Table S1. The average values of the concentration of Au ([Au]) found in tissues/blood at the end of study. Values are given in μg of Au detected per gram of organ and figures in brackets correspond to [Au] at Day 0.

Tissues	Average [Au] IV (μg/g)	Average [Au] IT (μg/g)
Liver	28.11 (0.791)	0.786 (0.078)
Spleen	59.423 (6.841)	1.073 (0.845)
Kidneys	0.801 (1.132)	0.025 (0.105)
Lungs	0.477	0.011
Heart	0.491	0.007
Brain	0.015	0.001
Tumor	2.609 (3.205)	10.083 (103.863)
Blood	0.015 (20.573)	0.004 (0.297)

Supplementary Table S2. Average blood results after 30 days. a, for mice receiving IV GNRs + PTT. b, for mice receiving IT GNRs + PTT group at the end of the study.

a

Biochemistry test	Results	Normal range
Urea	6.8 mg/dl	1.8-7.5
Creatinine	38 mg/dl	30-50
AST	215 IU/l	50-215
ALT	68 IU/l	27-78
ALP	60 IU/l	34-127

b

Hematology test	Results	Normal range
Hemoglobin	14.6 g/dl	12.7-18.4
White Blood Cells (WBC)	7.11 x 10^3 μl	3.10-13.04
Red Blood Cells	10.12 x 10^6 μl	8.83-11.72
Hematocrit	0.4621	0.45-0.66
Platelets	1043 x 10^3 μl	558-1564
WBC Differential	Normal	Normal

Supplementary Table S3. Mouse weight gain. The minimum, median, maximal and 1st and 3rd quartiles of weight gained by mice in each treatment arm of the study.

	IT GNRs + PTT	IV GNRs + PTT
Min (g)	1	1
Q1 (g)	1.25	3
Median (g)	3	4.5
Q3 (g)	4	5
Max (g)	6	6

Supplementary Table S4. ζ-potential of GNRs measured during synthesis and functionalization.

	**Zeta Potential** (mV)
GNR-CTAB	+ 62.8 ± 4.0
GNR-PEG-COOH	- 3.0 ± 1.5
GNR-PEG-Anti EGFR	-11.6 ± 1.2

Supplementary Table S5. PTT control parameters. The parameters of GNRs, and laser used in the control groups, with the tumor sizes measured at the start and end dates of the control arm. * This mouse received double the regular volume of IV GNRs.

**Control method**	**Vol. GNR (μl)**	**Conc. (GNR) nM**	**Laser Power (W)**	**Beam diameter (mm)**	**Laser time (min)**	**Tumor at day 0 (mm)**	**Tumor size at end (mm)**	**No. of days**
Laser only 1	-	-	1.00	6	3	5.5x5.1	14.5x11.4	30
Laser only 2	-	-	1.15	5	3	5.4x5.0	14.3x8.3	17
Laser only 3	-	-	2.20	8	3	5.8x4.9	6.0x5.5	30
IV GNRs – 1	100	5.50	-	-	-	5.5x4.3	11.3x9.8	17
IV GNRs – 2	200*	5.50	-	-	-	5.5x5.3	12.0x10.5	20
IT GNRs – 1	50	2.75	-	-	-	6.0x4.6	10.3x8.6	17
IT GNRs – 2	50	2.75	-	-	-	8.3x5.5	16x7.6	23

Supplementary Table S6. The parameters for the ten mice which received IV GNRs and PTT. The parameters of GNRs, and laser used in the IV PTT group, with the tumor sizes measured at the start and end dates.

**Mouse No.**	**Tumor size (mm) at Day 0**	**Vol. GNR (μl)**	**Conc. (GNR) nM**	**0D (GNR)**	**Laser Power (W)**	**Beam Diam. (mm)**	**Laser time (min)**
1	6.6 x 4.6	100	5.50	26.4	1.7	7.0	3
2	7.5 x 6.0	100	5.50	26.4	2.0	8.0	3
3	6.2 x 7.0	100	5.50	26.4	2.5	8.5	3
4	6.6 x 5.0	100	5.50	26.4	2.1	7.0	3
5	7.5 x 7.0	100	5.50	26.4	2.5	8.0	3
6	9.6 x 7.2	100	5.50	26.4	2.6	10.0	3
7	5.0 x 6.2	100	5.50	26.4	1.7	6.5	3
8	6.5 x 4.7	100	5.50	26.4	1.7	7.0	3
9	5.8 x 6.3	100	5.50	26.4	1.7	6.5	3
10	5.7 x 4.3	100	5.50	26.4	1.7	6.5	3

Supplementary Table S7. The parameters for the ten mice which received IT GNRs and PTT. The parameters of GNRs, and laser used in the IT PTT group, with the tumor sizes measured at the start and end dates.

Mouse No.	Tumor size (mm) at day 0	Vol. GNR (μl)	Conc. (GNR) nM	0D (GNR)	Laser Power (W)	Beam Diam. (mm)	Laser time (min)
1	7.3 x 5.7	50	2.75	13.2	2.0	7.0	3
2	7.2 x 6.0	50	2.75	13.2	2.0	7.0	3
3	5.3 x 4.9	50	2.75	13.2	2.2	8.0	3
4	9.1 x 6.3	50	2.75	13.2	2.5	9.0	3
5	5.5 x 4.8	100*	2.75	13.2	1.6	6.0	130s
6	5.1 x 5.1	50	2.75	13.2	1.1	5.5	3
7	8.5 x 5.5	50	2.75	13.2	2.2	8.0	3
8	6.4 x 6.2	50	2.75	13.2	1.8	9.0	3
9	7.3 x 4.8	70	2.75	13.2	2.1	8.0	3
10	6.4 x 6.2	40	2.75	13.2	1.0	6.0	3
